# Disease Prevalence Matters: Challenge for SARS-CoV-2 Testing

**DOI:** 10.3390/antib10040050

**Published:** 2021-12-17

**Authors:** Chin-Shern Lau, Tar-Choon Aw

**Affiliations:** 1 Department of Laboratory Medicine, Changi General Hospital, Singapore 529889, Singapore; michael.lau@mohh.com.sg; 2 Department of Medicine, National University of Singapore, Singapore 119077, Singapore; 3 Academic Pathology Program, Duke-NUS Medical School, Singapore 169857, Singapore

**Keywords:** SARS-CoV-2, disease prevalence, orthogonal testing

## Abstract

While sensitivity and specificity are important characteristics for any diagnostic test, the influence of prevalence is equally, if not more, important when such tests are used in community screening. We review the concepts of positive/negative predictive values (PPV/NPV) and how disease prevalence affects false positive/negative rates. In low-prevalence situations, the PPV decreases drastically. We demonstrate how using two tests in an orthogonal fashion can be especially beneficial in low-prevalence settings and greatly improve the PPV of the diagnostic test results.

## 1. Introduction

The need for robust screening policies is essential in the current pandemic. This has yet to be achieved: one study [1] of 936 centers showed that universal COVID-19 testing for surgical patients was only recommended in 18.4% of centers, with not all centers advocating the routine testing of hospital staff. It is thus essential for clinicians to understand how best to implement COVID-19 screening programs. For example, in our country, since May 2021, all healthcare workers are routinely screened twice a week with rapid antigen tests [2]. Several factors are important to consider in the community screening of COVID-19: the turnaround time of test results, the sensitivity of the assay, the frequency of testing, and the prevalence of the disease in the population.

Many rapid SARS-CoV-2 tests (antigen, RT-PCR, and antibody tests) are now available to assist in the screening and management of COVID-19. Although rapid nucleic acid tests are available and desirable due to their greater analytical sensitivity, supply of these reagents may be rate limiting, and they are also more expensive than antigen testing [3]. Although some studies have questioned the use of point-of-care tests (POCTs) in screening for COVID-19 [4,5], they did not consider an increased testing frequency in their assessments. For COVID-19, several studies have already demonstrated that repeated population screening, even with a low-sensitivity test, can reduce the average infectiousness of individuals and limit viral spread [6,7,8]. In a recent report [7], antigen lateral flow immunoassays (LFIAs) (Sofia SARS antigen assay) used every 3 days had sensitivities similar to centralized RT-PCR testing in patients with COVID-19 (sensitivities remained >0.98 over a 14-day period). This concurs with the latest Centers for Disease Control and Prevention guidelines [9] that recommend confirmatory PCR testing may not be necessary if serial antigen testing is performed every three days/twice per week. Antibody POCTs have also demonstrated good performance. In our own evaluation [10], we showed that assay sensitivities of the Abbott/Roche antibody POCTs can be 78.1% one week after the first positive RT-PCR.

However, in addition to testing frequency, it is important to understand how prevalence influences the performance of these tests, especially in low-prevalence situations. In previous articles that explore diagnostic methods of COVID-19 testing [11,12,13,14,15,16,17], this important concept is not elaborated upon, or only explained in brief. Thus, in this article, we explain the concepts of PPV/NPV and the influence of disease prevalence. We also emphasize how using two tests in an orthogonal fashion can be especially beneficial in improving the PPV of the individual tests in such circumstances.

## 2. Sensitivity, Specificity, PPV, and NPV

**Figure 1:**
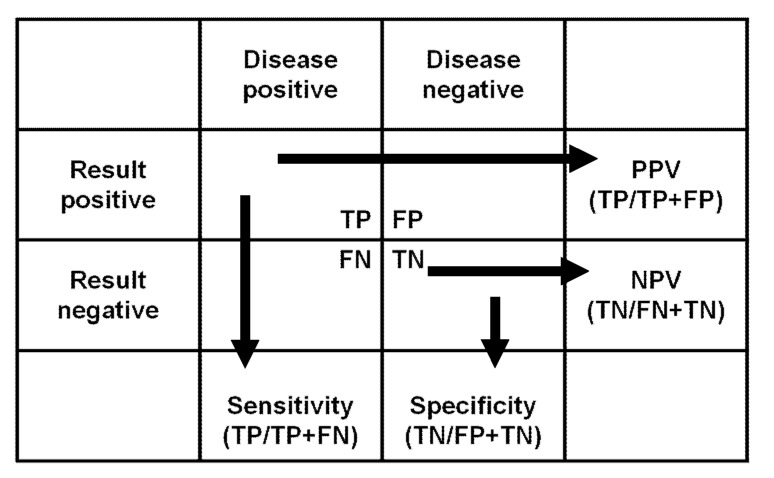
Overview of sensitivity, specificity, and positive and negative predictive values. Abbreviations: TP: true positive, FN: false negative, FP: false positive, TN: true negative, PPV: positive predictive value, NPV: negative predictive value.

There are several articles [18,19] that provide very good explanations for the concepts of assay sensitivity and specificity. Sensitivity represents how many diseased patients have a positive result. Specificity is the percentage of non-diseased patients with a negative test result. PPV is the probability of true disease when the result is positive. NPV is the probability of no disease when the result is negative (see Figure 1).

### The Effect of Disease Prevalence

The classic monograph [20] provides a clear explanation of the influence of prevalence on PPV/NPV. From the most recent Cochrane review [21], the SD Biosensor STANDARD Q COVID-19 rapid antigen test had a sensitivity/specificity of 69.2%/99.1%, with a PPV/NPV of 93.7%/93.3%, while a rapid molecular test (Cepheid Xpert Xpress) had a sensitivity/specificity of 88.1%/97.2%, with a PPV/NPV of 97.9%/98.4% (see Table 1). The performance of rapid antibody tests has improved greatly, with a recent study [22] stating that good lateral flow antibody assays with acceptable overall evaluations can have specificities of 98.0–99.5%, and sensitivities of 87.7–98.5%.

Applying these values to the readily available calculator from the Food and Drug Administration (FDA) [23], using a range of disease prevalence from 0.1 to 1.0%, we can obtain the corresponding PPV/NPV (see Table 2). Thus, when used alone, all three modalities will achieve a PPV of <10% when disease prevalence is 0.1%.

This means that in a population with a low disease prevalence, a positive result is confounded by a greater percentage of false positive cases. It is only when disease prevalence is at least 1.4%/3.1%/2.3% for the antigen/molecular/antibody POCTs that PPVs exceed 50%. This is especially important when testing the general population who are largely asymptomatic and when disease prevalence is low.

## 3. Orthogonal Testing

In low-prevalence settings, the high false positive results can be reduced by using two tests in an orthogonal fashion. In this situation, a second test is used to confirm the positive results from the first test. Only when both tests are positive is the final result considered truly positive. Calculations can be easily made on the FDA calculator [23]. In this way, when antigen testing is combined with either molecular or antibody testing in an orthogonal fashion at 0.1% prevalence, the PPV improves by 10- to 20-fold (see Table 2).

## 4. Discussion

Disease prevalence must be considered when interpreting test results. In a low-disease prevalence population, using one POCT on its own will generate more false positive results than when used in a higher-prevalence situation. This is especially challenging as the disease prevalence of COVID-19 varies greatly between and within populations. One recent meta-analysis [24] showed that the SARS-CoV-2 seroprevalence varied markedly among geographic regions, from 1.45% (South America) to 5.27% (Northern Europe). The difference in prevalence can occur even between different locations within the same country, as one UK study [25] showed that healthcare workers had an 11 times higher hazard ratio for COVID-19 than the general community. Tracking the prevalence of COVID-19 is also complicated by asymptomatic cases of COVID-19. In a survey of 936 centers across 71 countries [26], 27.5% of centers had experienced preoperatively asymptomatic patients testing positive only after surgery. Careful consideration of disease prevalence would be required before embarking on community testing, and screening programs may have to be more extensive in order to detect asymptomatic and pauci-symptomatic cases of COVID-19. When embarking on screening programs, medical centers would benefit from taking into account disease prevalence and orthogonal testing strategies.

However, in populations with a low disease prevalence, using two POCTs in an orthogonal fashion markedly improves the PPV of the results. The performance of orthogonal testing algorithms has already been reported. In a community COVID-19 seroprevalence study (*n* = 4333) [27], second-line testing (SARS-CoV-2 IgG spike antibodies, sensitivity/specificity 100%/98.4%) confirmed 78/98 (80%) initially positive test results (SARS-CoV-2 IgG nucleocapsid antibodies, sensitivity/specificity 96.4%/99.0%). The initial 2.3% seroprevalence was then revised downwards to 1.8%, proving that orthogonal testing can help reduce false positive rates. Another study [28] tested a population of seronegative Japanese healthcare workers (*n* = 1000) with a SARS-CoV-2 antibody POCT (Instant-view IgG/IgM Antibody COVID-19 LFIA) and had a positive rate of 3.3%. After applying a second automated antibody test (Roche Elecsys Anti-SARS-CoV-2 RUO assay) in an orthogonal fashion, all initially positive tests were considered false positives.

In summary, we wish to re-emphasize that population prevalence must always be considered when planning to implement SARS-CoV-2 population screening. In low-prevalence settings, the false positive rate may be unacceptably high, with disastrous consequences such as loss of confidence in the screening program, or over-use of resources from misclassification of cases. If community screening is planned, the use of two tests in an orthogonal fashion for cases with a positive first test is an important means to improve the PPV of COVID-19 screening: only cases with a positive first test are evaluated with a second test, and only when both tests are positive is the patient considered to have tested positive. As the second test is only used in those with a positive first test, this would be more cost efficient than performing two tests for all subjects.

## Figures and Tables

**Table 1:** Sensitivity, specificity, and predictive values of rapid tests.

Assay	Sensitivity	Specificity	PPV	NPV
Antigen (SD Biosensor)	69.2%	99.1%	93.7%	93.3%
Molecular (Cepheid)	88.1%	97.2%	97.9%	98.4%
Antibody *	87.7%	98.0%	-	-

* Values are the lower end of sensitivity and specificity from reference [22].

**Table 2:** Effect of prevalence and orthogonal testing on predictive values.

Parameter	Prevalence 0.1%		Prevalence 0.5%		Prevalence 1.0%	
	PPV	NPV	PPV	NPV	PPV	NPV
Single test						
Antigen	7.1	100	27.9	99.8	43.7	99.7
Molecular	3.1	100	13.7	99.9	24.1	99.9
Antibody	4.2	100	18.1	99.9	30.7	99.9
Orthogonal Testing						
Antigen → molecular	70.8	99.1	92.4	95.5	96.1	91.3
Antigen → Antibody	77.1	99.0	94.4	95.4	97.1	91.1

Abbreviations: PPV: positive predictive value, NPV: negative predictive value.

## Abbreviations

FDA: Food and Drug Administration; LFIA: lateral flow immunoassay; NPV: negative predictive value; POCTs: point-of-care tests; PPV: positive predictive value.
